# Estimation of the nutrient variation in feed delivery and effects on lactating dairy cattle

**DOI:** 10.3168/jdsc.2024-0564

**Published:** 2024-04-20

**Authors:** A.L. Carroll, K.J. Hanford, C. Abney-Schulte, P.J. Kononoff

**Affiliations:** 1 Department of Animal Science, University of Nebraska–Lincoln, Lincoln, NE 68583; 2 Department of Statistics, University of Nebraska–Lincoln, Lincoln, NE 68503; 3 Cargill, Minneapolis, MN 55440

## Abstract

Diets formulated for dairy cattle are designed to supply nutrients, but rations delivered to the animal may deviate from original formulations, resulting in implications for milk production, composition, and even pregnancy rate. The objective of this study was to evaluate retrospective feed mixing records collected from 8 commercial dairy farms over 52 wk. Farms ranged from 2,982 to 29,783 animals, producing 31.8 ± 5.93 (±SD) kg of milk with 4.14 ± 0.461% fat and 3.26 ± 0.218% protein. Data collected included daily TMR nutrient deviation, calculated as diet nutrient content minus the observed divided by the formulated diet concentrations of CP, fat, NDF, and starch. Data were analyzed with fixed effects of time (modeled as 28-d periods) and days of positive nutrient deviation in fat, CP, NDF, and starch with each nutrient alone and in combination with the other nutrients, random effects of herd, and repeated measure of time within herd. The nutrient deviation by farm averaged (±SD) 1.68 ± 2.655, 2.28 ± 4.473, 1.75 ± 2.621, and 1.47 ± 2.147% for CP, fat, NDF, and starch, respectively. Days of positive nutrient deviation per period averaged (±SD) 25.5 ± 3.47, 25.5 ± 3.72, 25.6 ± 3.49, and 25.9 ± 2.61, respectively. Dry matter intake (DMI; 17.2 ± 1.78 kg/d) decreased with increasing positive deviation days in starch (−0.0483 ± 0.01265) and increased with increasing positive deviation days in CP (0.0211 ± 0.00942). Milk yield (31.4 ± 2.50 kg/d) increased with positive deviation days in starch (0.0486 ± 0.02110) and decreased with increased positive deviation days in NDF (−0.0298 ± 0.02202). Finally, pregnancy rate (21.7 ± 4.34) increased with increasing positive deviation days in fat (0.385 ± 0.1635) and decreased with increasing positive deviation days in CP (−0.420 ± 0.1879). For these models, period significantly affected milk yield but was not observed to affect pregnancy rate and DMI. Results suggest farm and nutrient type were significant sources of variation, and overall, farms overfeed nutrients CP, fat, NDF, and starch 92% of the days of a 28-d period.

## Highlights

• Farms serve as a large source of variation in nutrient deviation.
• Farms overfeed nutrients 92% of the days within a 28-day period.
• Overfeeding CP may have negative implications on pregnancy rate.

## Graphical Abstract

**Figure:**
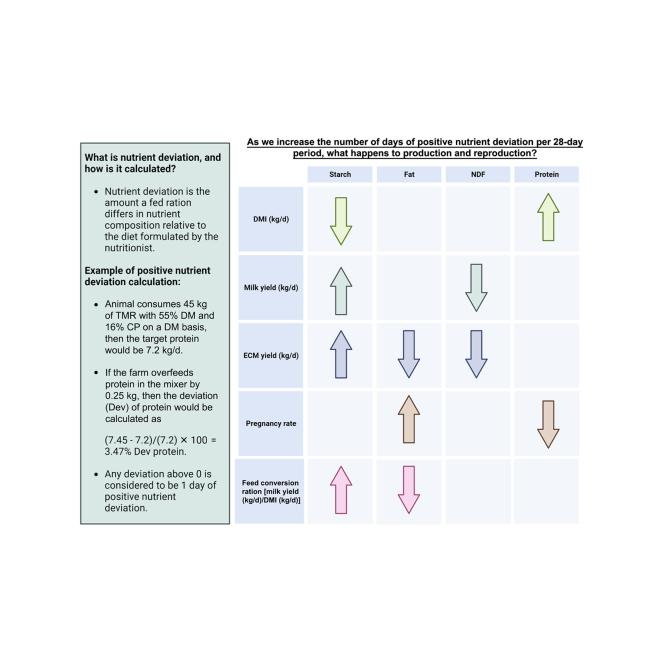
**Summary:** Recent advances in technology allow for an exchange of data between feeding equipment and nutritional management software. This allows for improved understanding of the differences between the formulated diet and the ration fed to the animals. The current experiment examined retrospective data from 8 dairies using a commercial software program (OneTrak Dashboard, Cargill Corn Milling, Blair, NE) to describe the effects of nutrient variation on production and pregnancy rate. Deviation of the fed ration from the formulated diet was evaluated for nutrients, including crude protein (CP), neutral detergent fiber (NDF), starch, and fat. Results suggest that farm and nutrient type were significant sources of variation, and overall, farms overfeed nutrients CP, fat, NDF, and starch 92% of the days of a 28-day period (n = 13). Practically, results indicate that overfeeding starch relative to dietary formulation may have a reducing effect on dry matter intake, and increasing positive deviation days in CP may have negative effects on pregnancy rate.

Diet formulation is the process of selecting individual feedstuffs and then blending them together in target proportions to supply nutrients needed to support the animal's maintenance and production (Patience, 2018). Nutrients supplied in a TMR delivered to a given group of cattle vary. Sources of this variation include inaccurate assumptions of the nutrient or moisture content of feedstuffs (Tran et al., 2020), contamination or deterioration of feed, and errors in weighing, mixing, and delivery of the TMR (Rossow and Aly, 2013; Trillo et al., 2016). Tebbe and Weiss (2020) noted that relative to a diet containing 95% of MP requirements (10.3% CP) oscillating CP every 48 h from 10.3% to 16.4% negatively influences milk composition. Additionally, when diets containing below and above computed requirements were offered in a daily alternating pattern, milk and milk protein responded to the daily changes in dietary protein. This observation suggests that cows require a continuous supply of MP. With the concern that milk production may be negatively affected by nutrient variation (Sova et al., 2014), practical tools have been developed to monitor daily feeding practices. An example is technology that calculates deviation in chemical composition in a mixed ration compared with that formulated (OneTrak Dashboard, Cargill Corn Milling, Blair, NE). The deviation from the original formulation is calculated from the weight of feeds loaded and their collective nutrient contribution. The objective of this study was to retrospectively analyze milk production and pregnancy rate records from commercial dairy farms, which measured nutrient deviation of a TMR from the original formulation. We hypothesized that increased days of positive nutrient deviation would negatively affect production and pregnancy rate.

Daily data were collected from 8 commercial dairies using a commercial software program (OneTrak Dashboard, Cargill Corn Milling), researchers were blinded to farm name and location and the breeds represented within the dataset included Holsteins, Jerseys, Ayrshires, and undisclosed crossbreds. Because the study used historical data collected from the field, the University of Nebraska–Lincoln Institutional Animal Care and Use Committee did not require approval of any animal procedures. Data used within the current experiment spanned November 2019 to November 2020. Measures included bulk milk tank measures of milk protein, fat, and overall milk volume. Nutrient deviation was automatically calculated from feed mixer data as the actual amount of a nutrient fed minus the target amount of the nutrient from the original diet formulation divided by the target amount of the nutrient from the original diet formulation. For example, if an animal is consuming 45 kg of a TMR at 55% DM and 16% CP on a DM basis, then the target protein would be 7.2 kg/d. If the farm overloads protein by 0.25 kg, then deviation of protein would be calculated as follows: (7.45 − 7.2)/(7.2) × 100 = 3.47%. Nutrient deviation was calculated in this manner for protein, fat, starch, and NDF for all lactating groups within each farm. The pregnancy rate was calculated as the number of animals pregnant divided by those that were eligible to be bred rather than a 21-d pregnancy rate. Data were collected from daily measurements of nutrient deviation and milk production and results were averaged for a 28-d interval (period) to represent one month in time. Days of positive nutrient deviation were counted as the days within the period where deviation was greater than zero and negative days were considered as those less than zero.

Data were analyzed in a repeated measures design GLIMMIX procedure of SAS (version 9.4, SAS Institute Inc.) with fixed effects of days of positive nutrient deviation per period alone or in combination with the other nutrients, and random effects of farm. Repeated measures included period and the covariance structure used was first-order autoregressive homogeneous. Model fit was evaluated with the Bayesian information criterion (**BIC**) with the lowest BIC being the best fit. If the BIC differed by small amounts of 0 to 2, then the simplest model was chosen (Raftery, 1995). This is because small differences within BIC values may have been present, and thus slight differences in BIC may not represent significant differences between the models.

The aim of this experiment was to use historical records and evaluate the influence of nutrient deviation in the delivered TMR relative to the nutritionist-formulated diet on milk production and pregnancy rate on commercial dairy farms. Table 1 lists the mean temperature-humidity index (**THI**; 66.0 ± 13.35) and calculated deviation in CP (1.68 ± 2.655%), fat (2.28 ± 4.473%), NDF (1.75 ± 2.621%), and starch (1.47 ± 2.147%) across 8 dairies. In the current analysis there was a large variation in the THI, particularly for farm ID 6. While we were blinded to farm location, this observation is unsurprising as THI can vary from 39.3 to 69.5 within the United States from winter to summer (Guinn et al., 2019). The nutrient with the largest range between farms during the 28-d period for nutrient deviation was fat ranging from 0.476% to 8.41%. This response was largely driven by increased fat deviation within a singular farm (farm ID 1); the variation in fat is not surprising because variation in nutrients can be farm dependent (Sova et al., 2014) based on ingredients chosen for the TMR, use of prepackaged supplements, and the practices of feed mixing personnel.

**Table 1:** Average period temperature-humidity index (THI) and nutrient deviation (%) of protein, fat, NDF, and starch by farm (mean ± SD)

Farm ID	Average THI and monthly nutrient deviation by farm				
	THI^1^	DevPro^2^	DevFat^2^	DevNDF^2^	DevStarch^2^
1 (n = 13)	64.6 ± 11.20	5.04 ± 0.691	8.41 ± 2.954	4.79 ± 0.970	2.94 ± 1.757
2 (n = 13)	67.2 ± 9.68	3.39 ± 2.943	1.91 ± 1.364	1.42 ± 1.355	1.05 ± 0.628
3 (n = 13)	66.2 ± 11.03	0.902 ± 0.1529	1.36 ± 0.350	1.16 ± 0.217	0.520 ± 0.163
4 (n = 13)	65.7 ± 10.15	0.367 ± 0.3180	1.02 ± 0.368	0.446 ± 0.3888	1.12 ± 0.338
5 (n = 12)	66.4 ± 10.08	1.52 ± 0.464	1.95 ± 0.851	1.51 ± 0.574	1.35 ± 0.533
6 (n = 12)	54.2 ± 18.44	1.36 ± 0.616	1.55 ± 0.731	1.91 ± 1.726	1.78 ± 0.947
7 (n = 13)	67.8 ± 9.60	1.05 ± 0.692	0.972 ± 0.7195	1.48 ± 0.914	0.749 ± 0.739
8 (n = 13)	67.0 ± 10.07	0.066 ± 0.8878	0.476 ± 0.9177	−0.059 ± 0.8863	0.423 ± 1.045
Mean	66.0 ± 13.35	1.68 ± 2.655	2.28 ± 4.473	1.75 ± 2.621	1.47 ± 2.147

1 Data collected from weather stations.

2 Nutrient deviation was automatically calculated from feed mixer data as the actual amount of a nutrient fed minus the target amount of the nutrient from the original diet formulation divided by the target amount of the nutrient from the original diet formulation. DevPro = deviation in protein; DevFat = deviation in fat; DevNDF = deviation in NDF; DevStarch = deviation in starch.

For each period, the number of days of positive nutrient deviation were summed and then analyzed by mean and SD. The number of days of positive nutrient deviation in each 28-d period across farms and average deviation for CP, fat, NDF, and starch was 25.5 ± 3.47 d, 25.5 ± 3.72 d, 25.6 ± 3.49 d, and 25.9 ± 2.61 d, respectively (Table 2). Within the current experiment, the count of the number of days of positive nutrient deviation was used as the dependent variable in the model. Data were analyzed as 28-d periods as daily data averaged similarly and caused reduced model convergence, which limits the understanding of the influence of the nutrients on the production measures. Although not evaluated in the current analysis, we recognize that the calculated nutrient deviation of an overall TMR is an oversimplification because individual feedstuffs such as corn silage have daily variation in nutrient composition (St-Pierre and Weiss, 2015). The practice of overfeeding nutrients has long existed because many diet formulations are designed to include so-called “margins of safety.” This is seemingly practiced to anticipate variability in feedstuffs and as a protective measure against underfeeding (Pesti and Miller, 1993). In the current study, farms overfed each nutrient approximately 26 d of the 28-d period which is equivalent to 92% of the time, and all nutrients were overfed a similar percentage of time ranging from 91.1% for CP at 25.5 d to 92.5% at 25.9 d for starch. This may imply some degree of heterogeneity in the ingredients that are overfed. More specifically, TMR ingredients are generally used to contribute to the requirements of one or a few nutrients within the diet; therefore, if a singular ingredient was overfed it may be observed in a few specific nutrients. Although diets are formulated to meet the requirements of the animal, this requires mixtures of differing ingredients. For example, when soybean meal is overfed it may cause increased CP deviation, whereas alfalfa hay may increase both NDF and CP deviation. Previous studies have indicated that ingredients such as corn silage, alfalfa hay, and canola meal are fed with low accuracy potentially as a function of ingredient distance from mixer and physical form (Trillo et al., 2016). Interactions between accuracy of weighting, nutrient composition, and physical form of the feedstuff likely exist and may play a role in the responses observed in the current study.

**Table 2:** Average days of positive nutrient deviation per period by farm (mean ± SD)

Farm ID	Average monthly number of positive deviation days by farm^1^			
	DevPro	DevFat	DevNDF	DevStarch
1 (n = 13)	27.9 ± 0.28	27.8 ± 0.38	27.9 ± 0.28	27.7 ± 0.85
2 (n = 13)	25.5 ± 4.12	25.5 ± 4.29	25.1 ± 4.72	24.6 ± 3.80
3 (n = 13)	27.7 ± 0.48	27.5 ± 0.97	27.6 ± 0.77	27.0 ± 1.22
4 (n = 13)	22.4 ± 4.03	25.5 ± 1.90	21.1 ± 5.01	26.4 ± 1.61
5 (n = 12)	27.3 ± 0.65	27.3 ± 0.62	27.6 ± 0.51	26.5 ± 1.45
6 (n = 12)	20.8 ± 3.28	17.7 ± 2.96	23.4 ± 1.83	22.7 ± 3.20
7 (n = 13)	26.8 ± 1.17	27.2 ± 0.73	27.1 ± 0.76	26.8 ± 1.28
8 (n = 13)	25.1 ± 2.53	25.0 ± 2.48	25.0 ± 2.58	25.0 ± 2.38
Mean	25.5 ± 3.47	25.5 ± 3.72	25.6 ± 3.49	25.9 ± 2.61

1 Nutrient deviation was automatically calculated from feed mixer data as the actual amount of a nutrient fed minus the target amount of the nutrient from the original diet formulation divided by the target amount of the nutrient from the original diet formulation. DevPro = deviation in protein; DevFat = deviation in fat; DevNDF = deviation in NDF; DevStarch = deviation in starch.

Table 3 lists models used to describe the influence of days of positive nutrient deviation on a given animal response. Model fit was assessed with the BIC where the lowest BIC represents the best fit (Brewer et al., 2016), and in interpreting the data the discussion will include the nutrients represented within the best fit model, and effect of 28-d period where significant. In the current study, animals in all stages of lactation were compiled together; therefore, our observations are limited to general effects and not specific to stage of lactation. Also, changes in ration formulations were not described within the data; however, they would have been accounted for within the calculated nutrient deviation. Although positive deviation days are presented, negative days deviation would be the inverse of the slope of positive nutrient deviation days. Dry matter intake (17.2 ± 1.78 kg/d) decreased with increasing positive deviation days in starch (−0.0483 ± 0.01265) and increased with increasing positive deviation days in CP (0.0211 ± 0.00942). Dry matter intake is a complex interaction of physical and metabolic signals (NASEM, 2021). The reduction in DMI associated with starch was expected because when starch is fermented in the rumen it supplies energy resulting in hypophagia via increased energy availability leading to decreased meal size (NASEM, 2021). Although rumen pH was not determined in the current experiment, increased dietary starch may lower rumen pH or cause ruminal acidosis, leading to decreased DMI (Plaizier et al., 2008). Increasing dietary CP has previously been shown to be positively associated with DMI (Allen, 2000) and indeed we observed a positive influence of CP on DMI with every positive deviation day leading to 0.0211 kg more DMI per day. Milk yield (31.4 ± 2.50 kg/d) and ECM (36.7 ± 2.49) increased with positive deviation days in starch (0.0486 ± 0.02110, 0.145 ± 0.0328) and decreased with increased positive deviation days in NDF (−0.0298 ± 0.02202, −0.0421 ± 0.02086). While starch negatively affected DMI, starch provision had positive influences on milk yield and ECM yield. Starch provides both propionate from bacterial degradation and glucose, thus providing energy to support lactation. A primary feedstuff within the formulated diets and fed rations was concentration of proprietary feed co-product containing wet corn gluten feed (OneTrak, Cargill Corn Milling). Wet corn gluten feed contains approximately 11.8% less NDF relative to corn silage (Ylioja et al., 2018), but has 20% to 23% more digestible NDF at the 48 h point in vitro relative to corn silage (NASEM, 2021). We speculate that since forages provide NDF within the diet that an increase in NDF may originate from a less digestible source. The resulting reduction in digestibility may then have had negative influences on milk yield and ECM. Interestingly, the number of positive deviation days in fat decreased (−0.143 ± 0.0427) ECM yield but was not identified as a significant factor in the milk yield equation. This may be a function of supplemental fat decreasing milk protein concentration with subsequent implications on protein yield, which is used within the ECM equation (Rabiee et al., 2012; NASEM, 2021). Another factor to consider when considering data over time is the influence of season on production. Period significantly affected milk yield and ECM negatively within periods corresponding to summer months of May, June, and July within the current dataset but was not observed as a significant effect within the DMI model. This was not expected as DMI is generally considered to be reduced during heat stress conditions (Wheelock et al., 2010). However, it should be noted that ∼35% to 50% of the reduction in milk yield during times of heat stress results from factors outside of the reduction in DMI (Rhoads et al., 2009; Wheelock et al., 2010). Feed conversion ratio within the current experiment was defined as the kilograms of milk production per animal divided by the kilograms of DMI. Feed conversion ratio as calculated as milk yield divided by DMI (1.95 ± 0.409) increased with increasing positive deviation days in starch (0.0149 ± 0.00548) and decreased with increasing positive deviation days in fat (−0.00312 ± 0.00688). The increased feed conversion ratio with increasing starch is expected as the numerator in the feed conversion ratio, milk yield, was also increased by increasing positive deviation days in starch. However, increasing positive deviation days in fat decreased feed conversion ratio. This would result from either decreasing the numerator of milk yield or increasing the denominator of DMI. Increasing fatty acids contain both unsaturated or saturated sources, but within the current analysis the exact fatty acid content of these diets cannot be determined. It has been previously observed that increasing saturated long-chain fatty acids increases DMI, which would decrease feed conversion if milk yield was not also improved (Weld and Armentano, 2017). Finally, pregnancy rate (21.7 ± 4.34) increased with increasing positive deviation days in fat (0.385 ± 0.1635) and decreased with increasing positive deviation days in CP (−0.420 ± 0.1879). The observations for the improved reproductive outcome with increased dietary fat is consistent with observations of Rodney et al. (2015) in which fat intake was reported to result in a positive impact on pregnancy risk at first service. However, it should be noted that the meta-analysis of Rodney et al. (2015) focused on transition animals and that calculation of pregnancy rate differed. In the current analysis pregnancy rate was defined as the number of animals pregnant divided by those that were eligible to be bred, whereas pregnancy at first service was used for pregnancy rate in the previous experiment by Rodney et al. (2015). The direct cause for improved reproduction with increased dietary fat is unclear (NASEM, 2021), but may be related to reduced negative energy balance and the positive influence of fat on reproductive tissue (Berry et al., 2016). The NASEM (2021, p. 96) states, “the current committee does not support the role of excess protein (i.e., to be beyond a reasonable safety factor) in the impairment of fertility.” It has been suggested that increased BUN is associated with feeding more protein, causing reduced pregnancy risk at first service (Lean et al., 2012), but this may also be a result of negative energy balance (Patton et al., 2014) and imbalances in metabolizable AA supply and not CP content per se.

**Table 3:** Influence of the count of positive days on DMI (kg/d), milk yield (kg/d), ECM (kg/d), pregnancy rate (%), and feed conversion ratio

Response	Average daily nutrient deviation (SEM, *P*-value)^1^					BIC^2^
	Intercept	DevStarch	DevFat	DevNDF	DevPro^1^	
DMI (kg/d)	17.2 (1.78, <0.01)	−0.0483 (0.01265, <0.01)			0.0211 (0.009418, 0.03)	275.3
Milk yield^3^ (kg/d)	31.4 (2.50, <0.01)	0.0486 (0.02110, 0.02)		−0.0298 (0.02202, 0.18)		340.7
ECM yield^3^, ^4^ (kg/d)	36.7 (2.49, <0.01)	0.145 (0.0328, <0.01)	−0.143 (0.0427, <0.01)	−0.0421 (0.02086, 0.05)		361.3
Pregnancy rate^5^	21.7 (4.34, <0.01)		0.385 (0.1635, 0.02)		−0.420 (0.1879, 0.03)	541.2
Feed conversion ratio^3^, ^6^	1.95 (0.409, <0.01)	0.0149 (0.00548, <0.01)	−0.00312 (0.00688, 0.65)			−36.0

1 Nutrient deviation was automatically calculated from feed mixer data as the actual amount of a nutrient fed minus the target amount of the nutrient from the original diet formulation divided by the target amount of the nutrient from the original diet formulation. DevPro = deviation in protein; DevFat = deviation in fat; DevNDF = deviation in NDF; DevStarch = deviation in starch.

2 BIC = Bayesian information criterion.

3 Significant effect of period (*P* < 0.01).

4 ECM calculated as (0.327 × milk, kg) + (12.95 × milk fat, kg) + (7.65 × milk protein, kg).

5 Pregnancy rate = number of animals confirmed pregnant/number of animals eligible to be bred.

6 Feed conversion ratio = milk yield (kg/d)/DMI (kg/d).

The aim of this experiment was to use retrospective records to describe the influence of nutrient deviation on measures of production and reproduction. Results indicate that farms can be a large source of nutrient variation between formulated diet and the ration between 28-d periods. We also observed that farms feed nutrients above the diet formulation 92% of the time. Overfeeding of starch relative to the formulated diets may lead to decreases in DMI. Our results suggest that fats may serve to improve energy density in these diets. Alternatively, increasing days of positive nutrient deviation in CP may have negative implications on pregnancy rate potentially stemming from unoptimized metabolizable AA. Overall, few experiments have aimed to test the influence of ration nutrient deviation in commercial conditions. Further analysis may allow for targeted alerts within feeding systems to improve feed management and consideration should be given to specific stages of lactation and parity. Therefore, research is needed in nutrient deviation to examine if parity and stage of lactation also affect the directionality and magnitude of the observed responses to nutrient deviation.
